# Carbon monoxide promotes stomatal initiation by regulating the expression of two *EPF* genes in Arabidopsis cotyledons

**DOI:** 10.3389/fpls.2022.1029703

**Published:** 2022-11-11

**Authors:** Xianjie Weng, Lingyan Zhu, Shuangshuang Yu, Yue Liu, Yanyu Ru, Zijing Zhang, Zhaorong He, Lijuan Zhou, Xiaolan Chen

**Affiliations:** ^1^ School of Life Sciences, Yunnan University, Kunming, Yunnan, China; ^2^ School of Agriculture and Life Sciences, Kunming University, Yunnan, China

**Keywords:** Arabidopsis thaliana, stomatal development, carbon monoxide, EPF2, STOMAGEN

## Abstract

The gaseous molecule carbon monoxide (CO) can freely pass through the cell membrane and participate in signal transduction in the cell to regulate physiological activities in plants. Here, we report that CO has a positive regulatory role in stomatal development. Exogenous CO donor CORM-2 [Tricarbonyldichlororuthenium (II) dimer] treatment resulted in an increase of stomatal index (SI) on the abaxial epidermis of cotyledons in wild-type, which can be reversed by the addition of the CO biosynthesis inhibitor ZnPPIX [Protoporphyrin IX zinc (II)]. Consistent with this result, mutation of the CO biosynthesis gene *HY1* resulted in a decrease of SI in *hy1-100* plants, while overexpression of *HY1* led to an increase of SI. Further investigation revealed that CO acts upstream of SPCH and YDA in the stomatal development pathway, since the loss of function mutants *spch-1* and *yda-2* were insensitive to CORM-2. The expression of *EPF2* was inhibited by CORM-2 treatment in wild type and is lower in *hy1* than in wild-type plants. In contrast, the expression of *STOMAGEN* was promoted by CORM-2 treatment and is higher in *HY1*-overexpression lines. Loss of function mutants of both *epf2* and *stomagen* are insensitive to CORM-2 treatment. These results indicated that CO positively regulates stomatal initiation and distribution by modulating the expression of *EPF2* and *STOMAGEN*.

## Introduction

Stomata are small pores in plants that mediate gas exchange on the epidermis of above ground organs such as the cotyledon, leaf, and stem. Stoma can respond quickly to multiple stimuli and its movement (opening and closing) affect the efficiency of both photosynthesis and respiration. In the dicotyledonous plant *Arabidopsis thaliana*, the formation of a stoma requires at least one asymmetric cell division (ACD) and one symmetric cell division (SCD), which is accompanied by a successive transition of cell fate (Pillitteri and Torii, 2012). Early in the development of young leaves, specific cells (meristemoid mother cells, MMCs) enter into the stomatal lineage and acquire the capability to divide asymmetrically, which produces two daughter cells, a small meristemoid (M) and a large SLGC (stomatal lineage ground cell). Meristemoids can undergo more ACDs (usually 1 to 3 times) to renew itself before the transition into a guard mother cell (GMC) (Nadeau and Sack, 2002). Finally, the GMC divide equally and produce two kidney-like guard cells (GCs) (Bergmann and Sack, 2007). Transcription factors, especially the basic helix-loop-helix (bHLH) family members, play core roles in stomatal development. SPEECHLESS (SPCH), MUTE, and FAMA (bHLH097) function in the successive divisions of stomatal lineage precursors, by forming heterodimers with ICE1/SCREAM or SCRM2 (Kanaoka et al., 2008). The SPCH/SCRMs regulates the ACD of MMCs and Ms and maintains their self-renewing capability (MacAlister et al., 2007). The MUTE/SCRMs module controls the transition of M to GMC and the exit of precursor from a stem cell identity (Pillitteri et al., 2007). The FAMA/SCRMs module regulates the terminal division of GMC and GC fate (Ohashi-Ito and Bergmann, 2006).

Distribution of stomata on the epidermis obeys the rule of “one-cell-spacing”,which is ensured by a complex cell-cell signaling pathway (Nadeau and Sack, 2002). Signaling peptides from EPFs (EPIDERMAL PATTERNING FACTOR) and CLVs (CLAVATAS/ESR-RELATED) families are secreted and perceived by receptors from ER (ERECTA) family and co-receptors TMM (TOO MANY MOUTHS) (Hara et al., 2007; Hara et al., 2009; Sugano et al., 2010; Lee et al., 2015; Vatén et al., 2018), which initiates a MAPK (MITOGEN-ACTIVATED PROTEIN KINASE) signaling cascade by phosphorylation. The MAPK cascade consists of the MAPKKK protein YDA(YODA), MAPKK protein MKK4/5/7/9, and the MAPK proteins MPK3 and MPK6. Activated MPK3/6 can phosphorylate SPCH and decrease its stability (Bergmann et al., 2004; Wang et al., 2007; Lampard et al., 2009). Members from the EPF family play vital roles in stomatal development. EPF1 and EPF2 secreted from epidermal cells act as a negative regulator, while STOMAGEN/EPFL9 from mesophyll acts as a positive regulator of stomatal density (Hara et al., 2009; Hunt and Gray, 2009; Sugano et al., 2010). EPF2 binds with ER-TMM and activates MAPK, which restricts the initiation of stomatal development by phosphorylating SPCH and decreasing its abundance (Bergmann et al., 2004; Lee et al., 2015). STOMAGEN competitively binds to ER-TMM to inhibit the activation of the MAPK cascade triggered by EPF2 (Lee et al., 2012; Lee et al., 2015). Thus, peptides from the same family fine-tune the density and patterning of stomata on the epidermis.

Carbon monoxide (CO), as a signaling molecule, functions in the regulation of multiple plant development processes and stress responses, such as lateral root formation (Guo et al., 2008; Chen and Kao, 2012; Samma et al., 2014), seed germination (Liu et al., 2007; Liu et al., 2010; Jia et al., 2018), cadmium tolerance (Han et al., 2008; Han et al., 2014), salt acclimation (Xie et al., 2011), stomatal movement (Cao et al., 2007; Song et al., 2008; Xie et al., 2016), and boron and iron homeostasis (Wei et al., 2011; Li et al., 2013; Yang et al., 2016; Lv et al., 2017). CO interacts with hormones and other signaling molecules to regulate the above processes. For example, CO promotes the accumulation of IAA during lateral root formation and dependent on the function of NO (Nitric Oxide) in tomato (Guo et al., 2008) and rice (Chen and Kao, 2012). CO can mediate ABA-induced closure of stomata in *Vicia faba* through interaction with NO and ROS (Cao et al., 2007; Song et al., 2008; Xie et al., 2016). In addition, crosstalk with NO, H_2_O_2_, and other small signaling molecules also play vital roles in CO-mediated stress responses to salt, drought, and heavy metals (Wang and Liao, 2016).

In plant cells, CO is mainly produced by heme oxidation, which is catalyzed by four HOs (heme oxygenase enzymes) in Arabidopsis (Muramoto et al., 2002; Emborg et al., 2006; Gisk et al., 2009). The four HOs are HY1(HO1), HO2, HO3, and HO4. HO1, encoded by HY1, can decompose heme and produce CO, as well as ions (Fe^2+^) and biliverdin IXa (BV) (Emborg et al., 2006; Gisk et al., 2009). BV is a precursor of the phytochrome chromophore, which participates in plant photomorphogenesis (Emborg et al., 2006).

Although broadly involved in multiple developmental processes, whether CO participates in the regulation of stomatal development remains unknown. Here we demonstrate that CO acts upstream of YDA to promote stomatal development in Arabidopsis cotyledons. Promotion of CO biosynthesis both endogenously and exogenously results in the increase of SI, while blocking of CO production chemically or genetically inhibits stomatal development. CO acts upstream of YDA to promote the stomatal index, as *yda-2* is insensitive to CORM-2 treatment. We also found that CO can modulate the expression of *EPF2* and *STOMAGEN*, and through their action regulate stomatal development.

## Results

### CO promotes stomatal development

**Figure 1:**
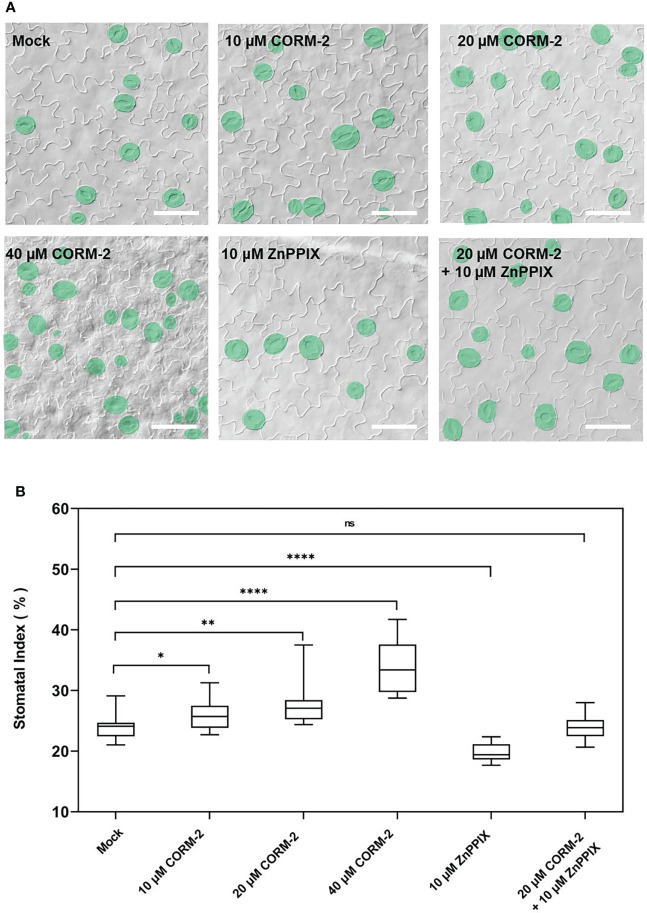
Exogenous CORM-2 treatment promotes stomatal development. **(A)** Differential interference contrast (DIC) images of the cotyledon epidermis of 14d old Wild-type (Col-0) plants with CORM-2 and ZnPPIX treatments. Bars = 50 μm. **(B)** Stomatal index (SI) of Wild-type (Col-0) with or without treatments. Error bars show standard deviation (SD) (n = 16). Statistical significance of different results was analyzed using Student’s *t*-test: ns Difference is not significant (*p*-value > 0.05). * Difference is significant (*p*-value < 0.05). ** Difference is highly significant (*p*-value < 0.01). **** Difference is highly significant (*p*-value < 0.0001).

To determine whether CO is involved in the regulation of stomatal density and distribution, Arabidopsis wild-type Columbia-0 (Col-0) seedlings were treated with CO donor CORM-2 at concentrations of 0, 10, 20, and 40 µM for 14 days (**Figure 1A**). We found that the stomatal index (SI) was significantly increased in treated seedlings compared with the control plants. The promotion effect of CORM-2 is concentration-dependent (**Figures 1A, B**). An increased SI (26.11±2.55)was found at a concentration of 10 μM compared with the control (24.09±1.90). The strongest promotion of SI was observed with 40 µM CORM-2 (33.91±1.90) (**Figure 1B**). Next, wild-type seedlings (Col-0) were treated with the HY1 inhibitor ZnPPIX to suppress CO production, which caused a significant decrease of SI in the wild type (19.87±1.37) (**Figures 1A, B**). Furthermore, ZnPPIX treatment also reversed the CORM-2-induced increase in SI (23.90±1.92) (**Figures 1A, B**). These results suggested that the CO donor treatment facilitated stomatal development.

**Figure 2:**
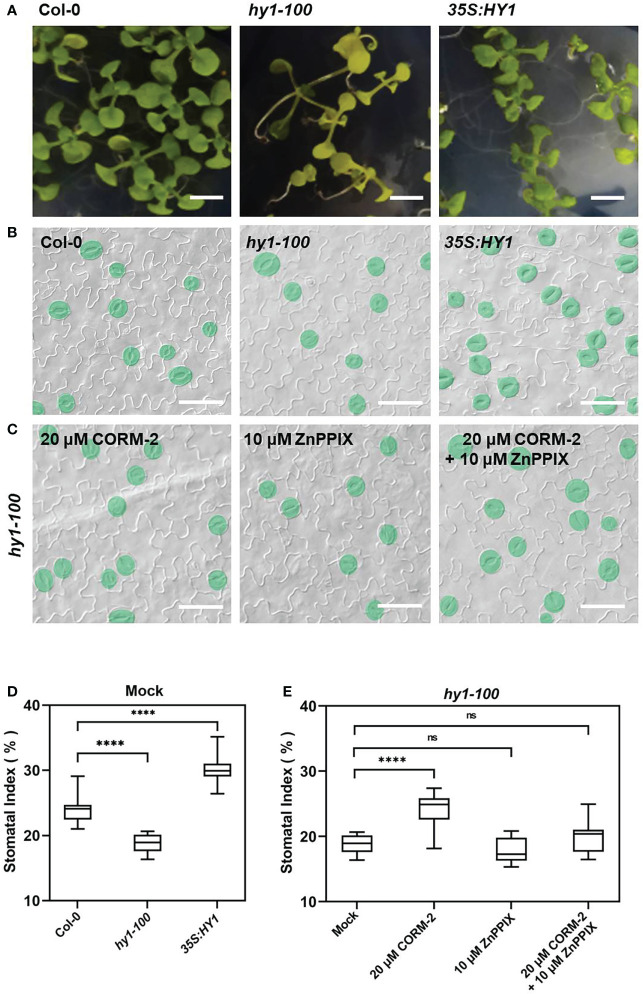
Stomatal development phenotype affected by loss-of (*hy1-100*) or overexpressed (*35S:HY1*) function of *HY1*. **(A)** Seedlings of 21d old of Wild-type (Col-0), *hy1-100*, and *35S:HY1* plants. Bars = 2 mm. **(B)** DIC images of the cotyledon epidermis of 14d old Wild-type (Col-0), *hy1-100* and *35S:HY1* plants. Bars = 50 μm. **(C)** DIC images of the cotyledon epidermis of 14d old *hy1-100* with CORM-2 (20 μM) and ZnPPIX (10 μM) treatment. Bars = 50 μm. **(D)** SIs of the Wild-type (Col-0), *hy1-100*, and and *35S:HY1* plants. Error bars show standard deviation (SD) (n = 16) **(E)** SIs of the *hy1-100* mutant treated with CORM-2 (20 μM), ZnPPIX (10 μM) and their combination treatments (20 μM CORM-2 and 10 μM ZnPPIX). Error bars show standard deviation (SD) (n = 16). Statistical significance of different results was analyzed using Student’s *t*-test: ns Difference is not significant (*p*-value > 0.05). **** Difference is highly significant (*p*-value < 0.0001).

**Figure 3:**
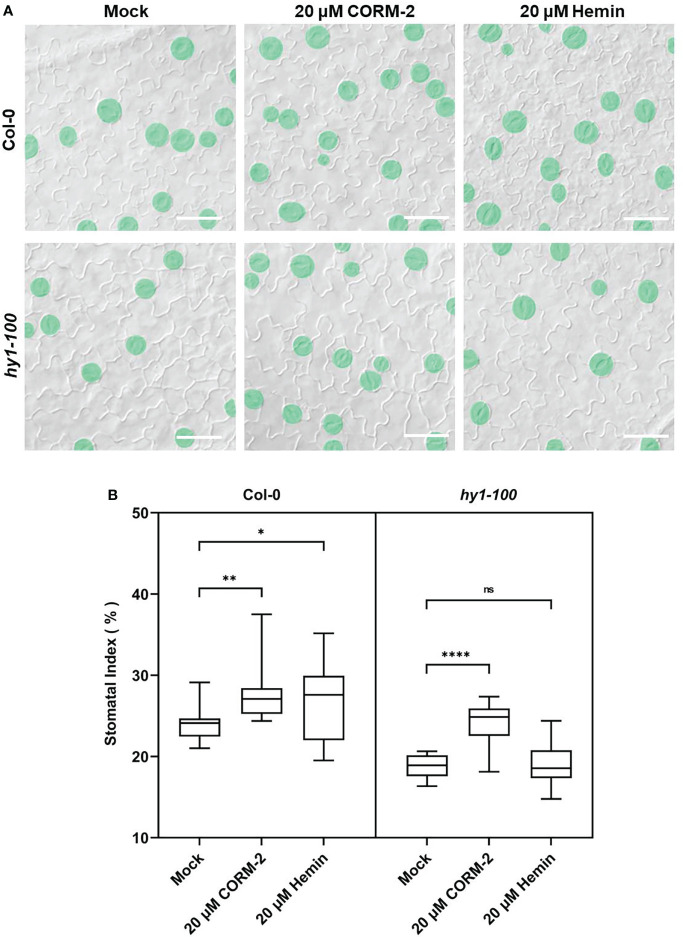
CORM-2 but not Hemin can restore the stomatal phenotype of *hy1-100*. **(A)** DIC images of the cotyledon epidermis of 14d old Wild-type (Col-0) and *hy1-100* plants with CORM-2 and hemin treatment. Bars = 50 μm. **(B)** SIs of Wild-type (Col-0) and *hy1-100* with or without CORM-2 (20 μM) and Hemin (20 μM) treatment. Error bars show standard deviation (SD) (n = 16). Statistical significance of different results was analyzed using Student’s *t*-test: ns Difference is not significant (*p*-value > 0.05). * Difference is significant (*p*-value < 0.05). ** Difference is highly significant (*p*-value < 0.01). **** Difference is highly significant (p-value < 0.0001).

Further experiments showed that endogenous CO has the same effects on the development of stomata. We examined the stomatal phenotypes of *hy1-100* (a CO-deficient mutant) and the *HY1-OE* line, which overexpressed *HY1* to generate higher endogenous CO (Xie et al., 2011; Yang et al., 2016). *hy1-100* is a loss of function mutant (CS236) which has an A to G substitution in the first intron (Muramoto et al., 1999), and no HY1 protein can be detected in this mutant (Xie et al., 2011). As previously reported, the mutation led to a longer hypocotyl (**Figure 2A**) in *hy1-100* homozygous plants than in wild type seedlings (Xie et al., 2011; Yang et al., 2016). We also found that the SI of *hy1-100* (18.85±1.42) was lower than that of wild type (24.09±1.90) (**Figures 2B, D**). In contrast, the SI was higher in *HY1-OE* transgenic plants (30.44±2.24) than in wild type (24.09±1.90) (**Figures 2B, D**). Most importantly, CORM-2 treatment effectively increased the SI in *hy1-100* to a level higher than the wild type, which can be effectively reversed by the addition of ZnPPIX (**Figures 2C, E**). ZnPPIX alone also led to a small decrease of SI in *hy1-100* (**Figures 2C, E**), which might be due to the presence of the other HOs in this mutant. In addition, treatment with hemin led to an increased SI in cotyledons of wild type, while it failed to induce the increase of SI in *hy1-100* seedlings (**Figures 3A, B**). These findings indicated that *HY1*-mediated hemin oxidation and CO biosynthesis positively affect stomatal development.

Since CO biosynthesis catalyzed by HY1 of hemin oxidation is accompanied by the production of Fe^2+^ and BV. Fe-EDTA and BV were used to treat wild type seedlings to detect whether they have any effect on stomatal development. There was not a significant difference between the Fe-EDTA, BV treatment and the control plants (**Figure S1**). These results precluded the possibility that the effect of HY1 on stomatal development is due to Fe^2+^ and BV, further proving that CO could promote stomatal development.

### CO regulates the expression of master regulators of stomatal development

**Figure 4:**
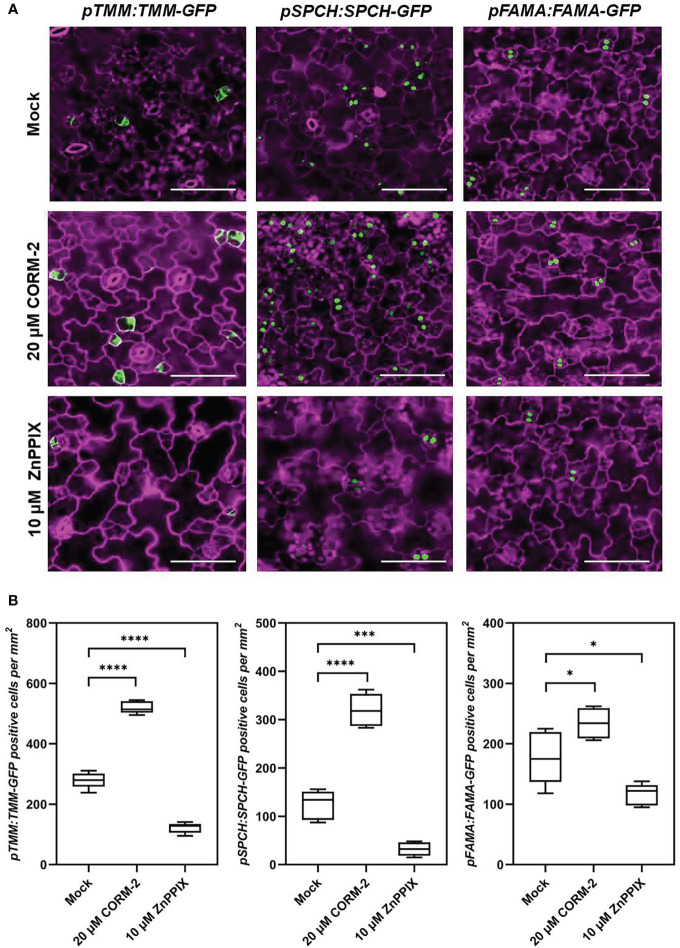
CO regulates the expression of key regulators of stomatal development. **(A)** Confocal images of the cotyledon epidermis of 4d old Wild–type (Col-0) plants expressing *pTMM:TMM-GFP* (*pTMM:TMM-GFP* in Col-0; first lane), *pSPCH:SPCH-GFP* (*pSPCH:SPCH-GFP* in Col-0; second lane), and *pFAMAp:FAMA-GFP* (*pFAMA:FAMA-GFP* in Col-0; third lane) with or without CORM-2 (20 μM) and ZnPPIX (10 μM) treatment. Bars = 50 μm. **(B)** Number of GFP-positive cells on the cotyledon epidermis of 4d old Wild–type (Col-0) plants expressing *pTMM:TMM-GFP*, *pSPCH:SPCH-GFP*, *and pFAMAp:FAMA-GFP* with or without CORM-2 (20 μM) and ZnPPIX (10 μM) treatment. Error bars show standard deviation (SD) (n = 5). Statistical significance of different results was analyzed using Student’s *t*-test: * Difference is significant (*p*-value < 0.05). *** Difference is highly significant (*p*-value < 0.001). **** Difference is highly significant (*p*-value < 0.0001).

To analyze how CO was involved in stomatal development, plants expressing a meristemoids specific reporter gene *pTMM:TMM-GFP* (Nadeau and Sack, 2002) was used for treatment. TMM-GFP-positive cells increased in the 20 µM CORM-2-treated seedlings (526.60±20.55 per mm^2^) while decreased in the 10 µM ZnPPIX-treated plants (121.60±17.30 per mm^2^) compared with the control (278.90±26.80 per mm^2^) (**Figures 4A, B**). Another reporter gene, *pSPCH:SPCH-GFP*, which expresses in the stomatal lineage precursor cells (MacAlister et al., 2007), was also treated with CORM-2 and ZnPPIX. Compared with the control (in which the number of cells expressing *pSPCH:SPCH-GFP* was 124.20±30.22 per mm^2^), more cotyledon epidermal cells (319.80±33.92 per mm^2^) expressed the marker *pSPCH:SPCH-GFP* in response to 20 µM CORM-2 treatment, while less cells (32.20±14.04 per mm^2^) in response to 10 µM ZnPPIX (**Figures 4A, B**). A third reporter, *pFAMA:FAMA-GFP*, which expresses in GMC and young GCs (Ohashi-Ito and Bergmann, 2006) was also used for treatment. We found that FAMA-GFP-positive cells also increased in CORM-2-treated cotyledons (234.0±25.18 per mm^2^) and decrease in ZnPPIX-treated cotyledons (116.20±17.80per mm^2^) than in controls (177.60±17.80 per mm^2^). These results show that exogenous CORM-2 can lead to the increase of the number of stomatal lineage cells on the cotyledon epidermis, while ZnPPIX has the opposite effect.

### CO acts upstream of the SPCH and YDA cascade to promote stomatal development

**Figure 5:**
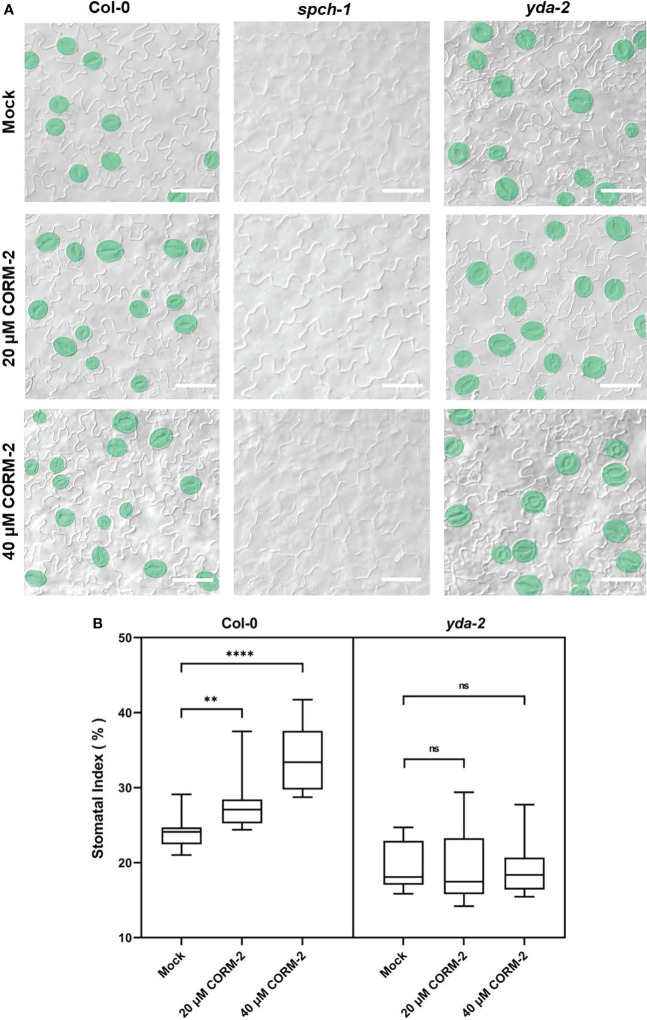
CO acts upstream of SPCH and YDA during stomatal development. **(A)** DIC images of the abaxial cotyledon epidermis of 14d old Wild–type (Col-0), *spch-1*, and *yda-2* plants with or without CORM-2 (20μM or 40μM) treatment. Bars = 50 μm. **(B)** SIs of Wild–type (Col-0), *spch-*1, and *yda-2* plants with or without CORM-2 (20μM or 40μM) treatment. Error bars show standard deviation (SD) (n = 16). Statistical significance of different results was analyzed using Student’s *t*-test: ns Difference is not significant (*p*-value > 0.05). ** Difference is highly significant (*p*-value < 0.01). **** Difference is highly significant (*p*-value < 0.0001).

The bHLH transcription factor SPCH is needed for the first entry division of stomatal lineage precursors. Without SPCH, as in the loss of function mutant *spch-1*, no cell can enter into the stomatal lineage leading to the formation of an epidermis with only pavement cells (MacAlister et al., 2007). The promotion of stomatal initiation by CO also needs the function of SPCH, as both CORM-2 and ZnPPIX treatment cannot rescue stomata formation in the *spch-1* mutant background (**Figure 5A**), which also suggested that CO functions upstream of SPCH to promote the initiation of stomatal divisions.

YDA is an upstream regulator of SPCH. It encodes for a MAPKKK protein in the MAPK cascade in stomatal development. YDA can integrate environmental factors with stomatal initiation by phosphorylation of SPCH through its downstream targets MPK3 and MPK6 (Gudesblat et al., 2012; Kim et al., 2012). To detect whether the promotion of stomatal development by CO is dependent on the function of YDA-initiated MAPK signaling, a loss of function mutant of *yda-2* was treated with CORM-2 and ZnPPIX. The results showed that *yda-2* was insensitive to CORM-2 and ZnPPIX. As shown in **Figure 5B**, no significant difference of SI was found in *yda-2* cotyledons in the presence of CORM-2 and ZnPPIX. These results indicated that CO regulated stomatal development in a manner dependent on the YDA-initiated pathway and functions upstream of the YDA cascade.

**Figure 6:**
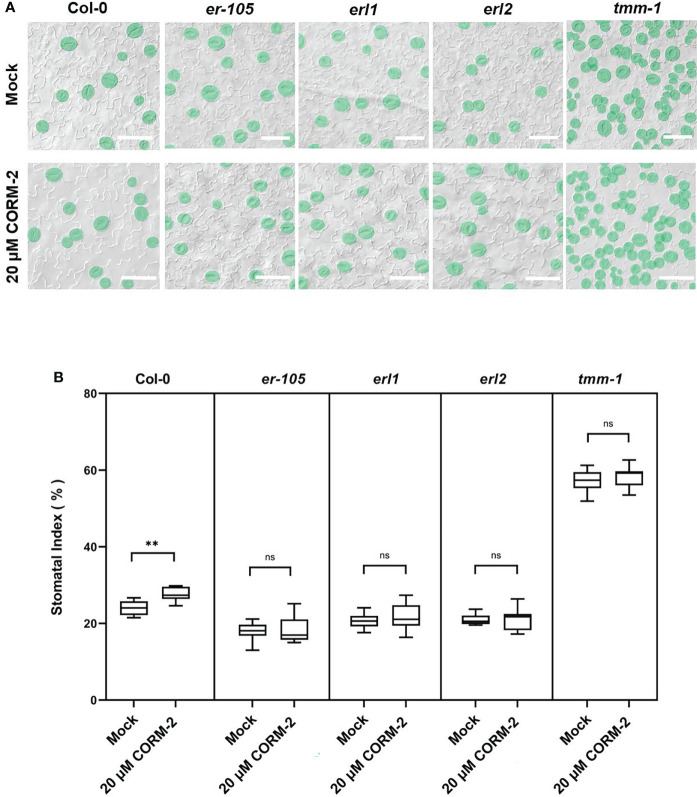
Response of Col, *tmm-1, er-105*, *erl1*, and *er2* seedlings to CORM-2 treatment. **(A)** DIC images of the abaxial cotyledon epidermis of 14d old Wild–type (Col-0), *tmm-1*, *er-105*, *erl1*, and *erl2* plants with or without CORM-2 (20 20) treatment. Bars = 50 μm. **(B)** SIs of Wild–type (Col-0), *tmm-1, er-105*, *erl1*, and *erl2* plants with or without CORM-2 (20 μM) treatment. Error bars show standard deviation (SD) (n = 8). Statistical significance of different results was analyzed using Student’s *t*-test: ns Difference is not significant (*p*-value > 0.05). ** Difference is highly significant (*p*-value < 0.01).

TMM-ERs complexes are membrane receptor kinases which bind different ligands from outside and transduce the signaling into the cell though activating the YDA cascade (Nadeau and Sack, 2002; Lee et al., 2015). To detect whether TMM and ERs were required for CO to regulate stomatal development, loss of function mutants of *tmm-1*, *er-105*, *erl1*, and *erl2* were treated with CORM-2. CORM-2 treatment induced a slight, but significant increase of SI in *tmm1* at a concentration of 20 µM, which indicated that *tmm-1* was less sensitive to CORM-2 treatment compared with wild type (**Figures 6A, B**). The *er-105*, *erl1*, and *erl2* mutants were also less sensitive to CORM-2 treatment because their SIs were less affected by treatment than wild type (**Figures 6A, B**). The changed sensitivity of the above mutants to CORM-2 indicated that regulation of stomatal development by CO may depend on the function of TMM and ERs.

### CO regulates the expression of *EPF2* and *STOMAGEN*

**Figure 7:**
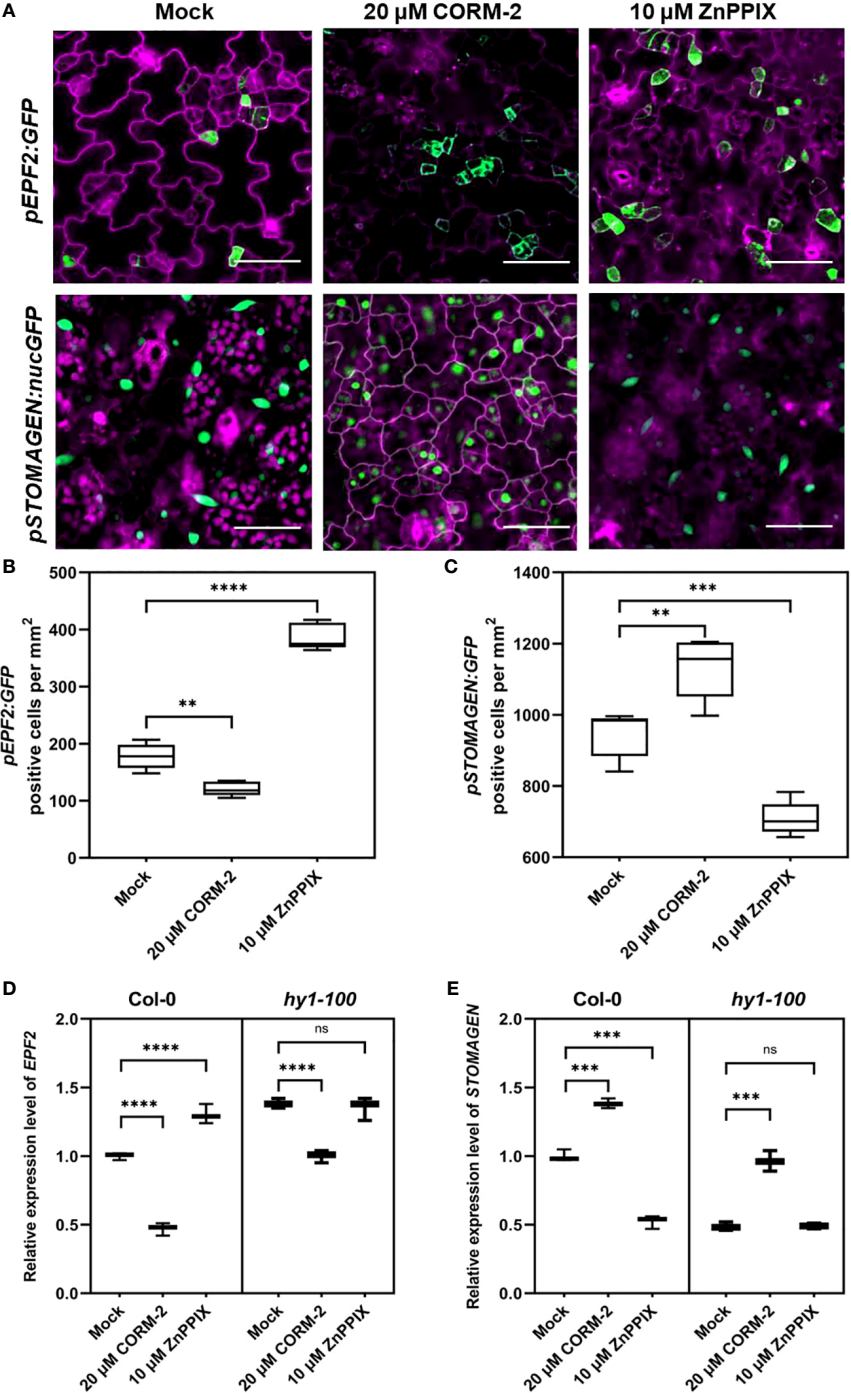
CO regulates the expression of *EPF2* and *STOMAGEN*. **(A)** Confocal images of the abaxial cotyledon epidermis of 4d old Wild–type (Col-0) harboring *pEPF2:GFP* and *pSTOMANGEN:nucGFP* with or without CORM-2 (20 μM) treatments and ZnPPIX (10 μM) treatment. Bars = 50 μm. **(B, C)** Number of GFP-positive cells on the cotyledon epidermis of 4d old Wild–type (Col-0) plants expressing *pEPF2:GFP* **(B)** and *pSTOMAGEN:nucGFP* **(C)**. Error bars show standard deviation (SD) (n = 5). **(D, E)** Relative mRNA level of *EPF2* **(D)** *and STOMAGEN* **(E)** in Wild–type (Col-0) and *hy1-100* seedlings (4dap) with or without CORM-2 (20 μM) treatments and ZnPPIX (10 μM) treatment. Error bars show standard deviation (SD) (n = 3). Statistical significance of different results was analyzed using Student’s *t*-test: ns Difference is not significant (*p*-value > 0.05). ** Difference is highly significant (*p*-value < 0.01). *** Difference is highly significant (*p*-value < 0.001). **** Difference is highly significant (*p*-value < 0.0001).

ER and ERL1 form a complex with TMM and specifically bind the peptides from the EPFs family to modify the density and distribution of stomata (Lee et al., 2015). Since TMM and ER/ERL1 may function in CO-regulated stomatal development, we asked whether the expression of *EPFs* was affected by CO. We used the promoters of *EPFs* to express a GFP marker (*pEPF1:GFP*, *pEPF2:GFP* and *pSTOMAGEN:nucGFP*) to detect the effect of CORM-2 and ZnPPIX on their expression. The expression of *pEPF2:GFP* was inhibited by CORM-2 and promoted by ZnPPIX treatment (**Figures 7A, B**). In contrast, there was no difference in *pEPF1:GFP* expression in the presence of CORM-2 and ZnPPIX (**Figures S2A, S2B**). Moreover, the expression of *pSTOMAGEN:nucGFP* increased after CORM-2 treatment and decreased after ZnPPIX treatment (**Figures 7A, C**).

Real-time PCR experiments confirmed the function of CO on the *EPFs* genes expression. A significant increase (by 38.33 %) of *STOMAGEN* and a decrease (by 53.00 %) of *EPF2* were found after CORM-2 treatment in wild type (**Figures 7D, E**). In contrast, no difference in the expression level of *EPF1* was seen with CORM-2 and ZPPIX treatment (**Figure S2B**). In accordance with these results, a lower mRNA level of *STOMAGEN* and a higher expression of *EPF2* were found in *hy1-100* than in wild type (**Figures 7D, E**). In addition, exogenous CORM-2 treatment can restore the expression of *STOMAGEN* and *EPF2* in *hy1-100* to wild type levels (**Figures 7D, E**). These results showed that CO can promote the expression of *STOMAGEN* and inhibit the expression of *EPF2*.

### CO promotes the stomatal development in a way dependent on EPF2 and STOMAGEN

**Figure 8:**
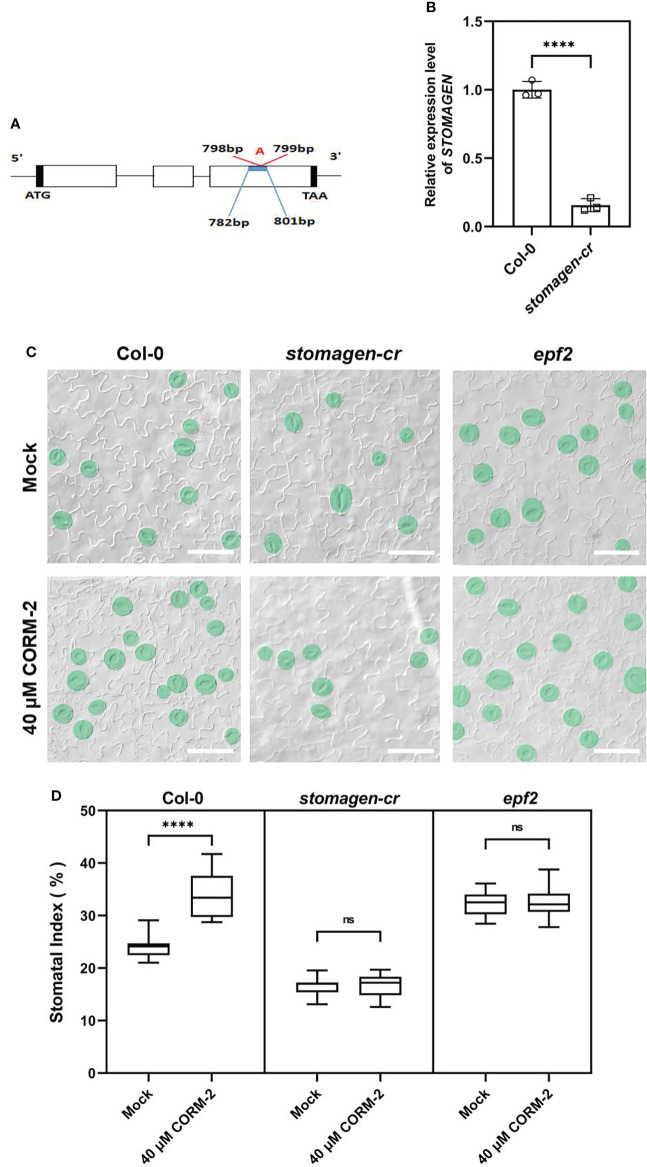
Regulation of stomatal development by CO is dependent on the function of EPF2 and STOMAGEN. **(A)** Insertion site of CRSIPR-Cas9 line of the *stomagen* mutant. **(B)** Expression level of *STOMAGEN* in Wild–type (Col-0) and *stomagen-cr* mutant produced by CRISPR-Cas9. Error bars show standard deviation (SD) (n = 3). **(C)** DIC images of the abaxial cotyledon epidermis of 14d old Wild–type (Col-0), *epf2*, and *stomagen-cr* plants with or without CORM-2 (40 μM) treatment. Bars = 50 μm. **(D)** SIs of Wild–type (Col-0), *epf2*, and *stomagen-cr* plants with or without CORM-2 (40 μM) treatments. Error bars show standard deviation (SD) (n = 16). Statistical significance of different results was analyzed using Student’s *t*-test: ns Difference is not significant (p-value > 0.05); **** Difference is highly significant (*p*-value < 0.0001).

Since CO can regulate the expression of *EPF2* and *STOMAGEN*, we then asked whether the promotion of stomatal development by CO was dependent on the function of EPF2 and STOMAGEN. First, a CRISPR/Cas9 system was employed to edit the *STOMAGEN* gene and produce a loss-of-function mutant. The homozygous *stomagen-cr* mutant contained a 1-bp insertion between 798 and 799bp of the coding region (**Figure 8A**), which caused a decrease of the mRNA level (by 84.34 %) (**Figure 8B**) and SI (by 7.58 %) on the cotyledon epidermis (**Figure 8C**), a phenotype similar to previously reported RNAi lines (Sugano et al., 2010). We then treated this mutant with 40 μM CORM-2, and found that it was insensitive to the chemical as no significant change of SI was found after treatment (**Figures 8C, D**). As well, an *epf2* loss of function mutant was also treated with 40 μM CORM-2 and the SI did not significantly change (**Figures 8C, D**). Taken together, these results verified that CO regulates stomatal development in a way dependent on the function of EPF2 and STOMAGEN.

## Discussion

### CO is a positive regulator of stomatal development

Mounting evidence indicates that CO can participate in a variety of developmental processes and stress responses in plants (Wang and Liao, 2016). For instance, CO positively affects seed germination (Liu et al., 2007; Liu et al., 2010; Jia et al., 2018) and lateral root formation (Guo et al., 2008; Chen and Kao, 2012; Samma et al., 2014), and protects plant against heavy metals (Han et al., 2008; Wei et al., 2011; Zheng et al., 2011; Yang et al., 2016), osmotic stress (Liu et al., 2010; Xie et al., 2011) and oxidative stress (Wu et al., 2010). Stomata are small pores through which plants exchange water and gases with the atmosphere, and both environmental factors and gaseous signaling molecules can modulate their development (Wu et al., 2010; Fu et al., 2016; Han et al., 2018; Deng et al., 2020). CO has been extensively reported to affects stomatal movement (She and Song, 2008; Song et al., 2008). However no reports focus on the effect of CO on stomatal development. In this study, we report that CO can promote stomatal initiation to increase stomatal index. Treatment with CORM-2 facilitates a dose-dependent increase of stomatal index (**Figures 1A-C**). CORM-2 treatment also promotes the expression of marker genes, including the MMCs- and Ms-expressed *pTMM:TMM-GFP* and *pSPCH:SPCH-GFP*, and GMC- and GC-expressed *pFAMA:FAMA-GFP*) (**Figure 4**). In contrast, treatment with the CO biosynthesis inhibitor ZnPPIX and a mutation of *HY1* that disrupted CO generation dampened stomatal development in Arabidopsis cotyledons (**Figures 1**, **2B**). In addition, the restoration of the capacity for CO generation through CORM-2 restored a wild type stomatal index in the *hy1-100* mutant plants (**Figures 2C, E**).

### Possible regulation mechanism of CO in stomatal development

In Arabidopsis, peptides from the EPFs family are perceived by membrane-located receptors to fine-tune the initiation and distribution of stomata (Lee et al., 2012; Jewaria et al., 2013; Lee et al., 2015). Environmental factors and hormonal signaling molecules regulate stomatal initiation by mediating the expression of EPFs (Engineer et al., 2014; Zhang et al., 2014; Hepworth et al., 2015; Caine et al., 2016; Wang et al., 2021). EPF2 is perceived by ER and its co-receptor TMM. Binding of EPF2 with the ER-TMM complex triggers rapid phosphorylation of the downstream of MAPK cascade (Bergmann et al., 2004; Lee et al., 2012; Lee et al., 2015). STOMAGEN can competitively bind to ER-TMM to replace EPF2, and diminish the downstream activation of MAPK elicited by EPF2 binding (Lee et al., 2015). In this study, we found that CO regulates the expression of *STOMAGEN* and *EPF2*. First, CORM-2 treatment downregulated *EPF2* expression and upregulated *STOMAGEN* expression in the wild type (**Figures 7B, C**). Secondly, the expression level of *EPF2* was higher in *hy1-100* than in the wild type (**Figure 7D**). A lower expression level of *STOMAGEN* in *hy1-100* compared to the wild type were also detected (**Figure 7E**). Third, the higher expression of *EPF2* and lower expression of *STOMAGEN* in *hy1-100*, were rescued by treatment with CORM-2 (**Figures 7D, E**). Fourth, loss of function mutants of *epf2* and *stomagen* were insensitive to CORM-2 treatment (**Figures 8C, D**), which confirmed that CO needs the functions of EPF2 and STOMAGEN to promote stomatal development. In conclusion, our data suggest that CO plays a positive role in the regulation of stomatal initiation in Arabidopsis cotyledons *via* modulating the expression of *EPF2* and *STOMAGEN*.

### HY1 is involved in the regulation of two pathways for stomatal development in Arabidopsis

**Figure 9:**
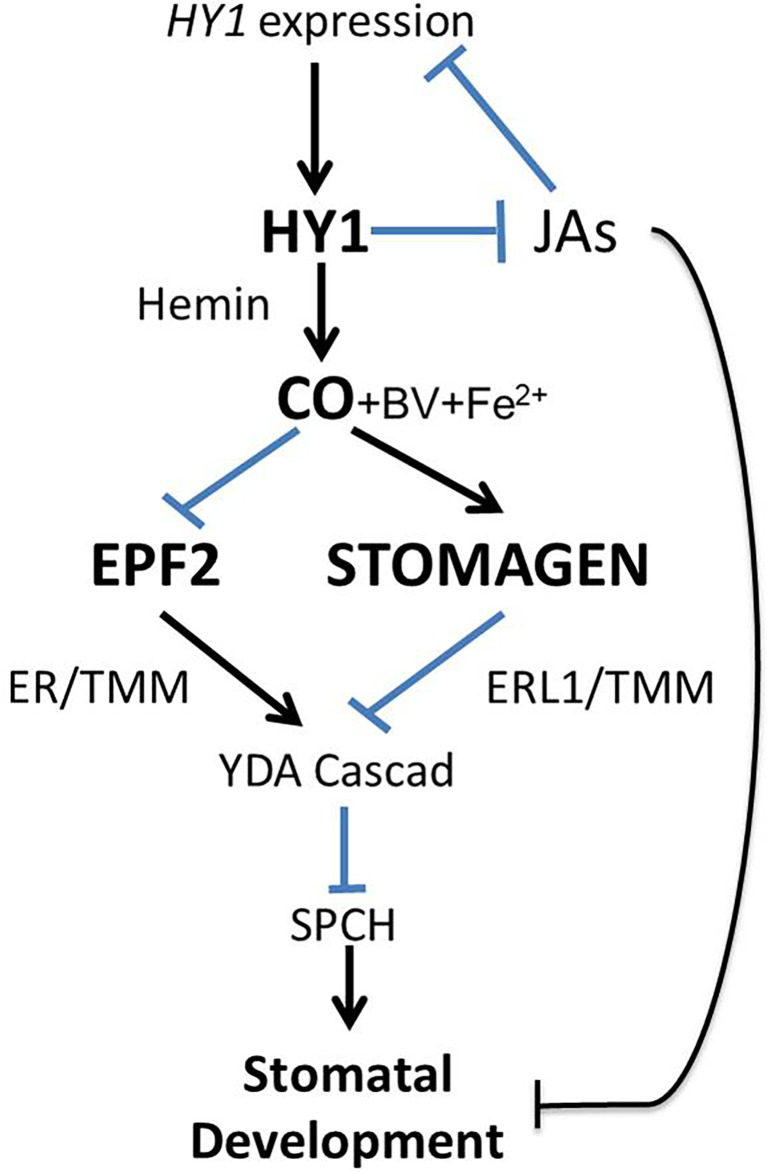
Model of the regulation mechanism of CO in stomatal development.

HY1 plays a crucial role in the endogenous production of CO, as HY1, encoding a heme oxygenase, degrades hemin producing Fe^2+^, BV, and CO. In this report, we precluded the possibility that the effect of HY1 on stomatal development is due to the byproducts Fe^2+^ and BV (**Figure S1**). Moreover, a previous study reported that the *hy1-100* mutant has a higher JA level than wild type (Zhai et al., 2007), and JAs negatively regulates stomatal initiation in Arabidopsis cotyledons (Han et al., 2018). Therefore, based on our results and these reports, we propose the possible regulation mechanism of HY1 catalyzed hemin oxidation in stomatal development as shown in **Figure 9**. On one hand, as a product of heme oxidation catalyzed by HY1 in the chloroplast, CO promotes the expression of *STOMAGEN* in mesophyll cells and also diffuses to epidermal cells where it inhibits the expression of *EPF2*. Less EPF2 from epidermal cells and more STOMAGEN synergistically release downstream inhibition of SPCH through the MAPK pathway. On the other hand, HY1 inhibits the accumulation of JAs and removes the inhibition of JAs in stomatal initiation. In addition, JAs inhibits the expression of *HY1* conversely (Zhai et al., 2007), which leads to the formation of a feedback loop between the production of JAs and HY1 activity. In the wild type, the formation of the feedback loop fine-tunes the expression level of SPCH and thus the stomatal production. In the *hy1* mutant, the block of this feedback resulted in low levels of CO and overaccumulation of JAs which synergistically inhibit the formation of stomata on the cotyledon epidermis. In our previous research, another gaseous signaling molecule, H_2_S, also regulates stomata through JA signaling (Deng et al., 2020). Therefore, JA signaling is important to regulate stomata development through gaseous signaling molecules. Nevertheless, the mechanism underlying the crosstalk of JA and CO/H_2_S remains largely unknown.

Moreover, CO is also involved in the regulation of darkness-induced NO synthesis (She and Song, 2008), and the crosstalk between CO and NO has been shown in seed germination regulation (Liu et al., 2010). As a positive regulator, NO can promote the expression of *SPCH*, *MUTE*, and *SCRM2* and inhibit the expression of *MPK6* (Fu et al., 2016). Furthermore, CO is also involved in IAA-regulated adventitious root development (Xuan et al., 2008). A previous study reported that the auxin-related transcription factor ARF5/MONOPTEROS (MP) can repress the expression of *STOMAGEN* by directly binding to its promoter (Zhang et al., 2014). While little is known about the cross talk between CO and auxin in stomatal development, it is reasonable to deduce that CO may regulate the expression of *STOMAGEN* through the *ARF5*-mediated auxin pathway, a hypothesis which requires further experimental verification. As well, further investigation is required to establish the relationship between CO and auxin through the EPF family, which will provide insights into the complicated regulatory mechanism governing the initiation of stomata.

## Materials and methods

### Materials and plant growth conditions

*Arabidopsis thaliana* plants were grown on half-strength Murashige and Skoog (MS) medium at 22°C under a 16-h light/8-h dark photoperiod. Chemicals were purchased from the Shanghai Sangon Biotechnology Co. Ltd. (Shanghai, China). COMR-2 and ZnPPIX were obtained from Sigma (St. Louis, Missouri, USA), and Taq DNA polymerases were from Takara Biotechnology (Dalian, China). The mutants used in this study are: *tmm-1* (Yang and Sack, 1995), *spch-1* (MacAlister et al., 2007), *yda-2* (CS6393, Lukowitz et al., 2004), *epf2* (Hara et al., 2007), and *35S:HY1* transgenic plants (Xie et al., 2011). Transgenic plants with stomatal lineage-specific markers (*pTMM:TMM-GFP, pSPCH:SPCH-GFP*, *pFAMA:FAMA-GFP, pEPF1:GFP*, and *pEPF2:GFP*) were obtained from Fred Sack (University of British Columbia). The mutants *hy1-100* (CS236), *er-105* (CS6588), *erl1* (SALK_081669C), and *erl2* (SALK_144166C) were from the Nottingham Arabidopsis Stock Centre (NASC).

### Chemical treatment

Stock solution (at 10 mM) of COMR-2 and ZnPPIX (Sigma) were dissolved in DMSO (dimethylsulphoxide). Final use concentration solutions were made by diluting stocks with sterilized medium. In this study, 10, 20, and 40 μM were used as final concentrations for CORM-2, and 10 μM for ZnPPIX. An equivalent concentration of DMSO was added in the plates as control. Seeds were sterilized and germinated on half-MS medium supplemented with or without chemicals. All the treatments have at least three replicates.

### Microscopy

An OLYMPUS BX51 microscope was used to take DIC images from samples which were stored in Hoyer’s solution. The 14 dag (day after germination) cotyledons were used for observation. The clearing of samples was as follows: first seedlings were immersed in 70% ethanol. After being cleared overnight at room temperature, samples were transferred into the Hoyer’s solution for storage. To observe the expression of marker genes, a ZEISS LSM800 laser confocal scanning microscope was used for shoot GFP fluorescence images and PI (propidium iodide) (Sigma-Aldrich) staining. Confocol images were cleared using magenta for the PI channel.

### Stomatal index measurement

An OLYMPUS BX51 microscope was used to visual the samples placed in water. Stomatal index (SI) is defined as the ratio of stomata and stomatal lineage precursors (MMC and M) in total epidermal cells per unit area. For stomatal index, number of stomata, stomatal lineage precursors (MMC and M) and all epidermal cells of 0.37 mm^2^ were counted to acquire a SI value for different cotyledons from different plants. Cotyledons from at least eight different plants were selected from the used genotypes.

### RNA extraction and qRT-PCR

Total RNA extraction and qRT-PCR were performed as described previously (Han et al., 2018; Deng et al., 2020). Trizol reagent (Invitrogen) was used to extract total RNA. For qRT-PCR, 1 μg DNase-treated RNA in 20 μl reaction volumes were used to synthesis the first-strand using M-MuLV reverse transcriptase (Monad, China) with oligo (dT)18 primer. 2×TB Green Premix Ex Taq II (Tli RNaseH) was used to perform PCR on an Applied Biosystems QuantStudio^TM^ 6 flex system real-time PCR machine, according to the manufacturer’s instructions. Three replicates for each sample were used for qRT-PCR analysis. The *Actin* gene was used as an internal control. Gene-specific primers used to detect transcripts are listed in **Supplemental Table S1**.

### Construction of plasmids

For the construction of *stomagen-cr* lines, target sites were designed by the use of CRISPR-GE to avoid off-targets (http://skl.scau.edu.cn/; Ma et al., 2015). The editing vectors were constructed as described in a previous report (Zhang et al., 2019). The sgRNA containing a single target site driven by the AtU3b promoter was cloned into the pMH-SA vector by the restriction enzyme sites AscI. Homozygous mutant lines were identified by sequencing.

For the construction of *pSTOMAGEN:nucGFP*, the 2,000 bp promoter sequence of AT4G12970 (*STOMAGEN*) was amplified by PCR and cloned in front of nucGFP in the Poca30 binary vector. The primers and restriction enzyme sites used to amplify sequences and generate vectors are listed in **Supplemental Table S1**.

### Accession numbers

Arabidopsis Genome Initiative numbers for the genes used in this article are as follows: *HY1* (AT2G26670); *TMM* (AT1G80080); *ER* (AT2G26330); *ERL1* (AT5G62230); *ERL2* (AT5G07180); *YODA* (AT1G63700); *SPCH* (AT5G53210); *FAMA* (AT3G24140); *EPF1* (AT2G20875); *EPF2* (AT1G34245); *STOMAGEN* (AT4G12970) and *ACTIN (*AT3G18780).

## Data availability statement

The original contributions presented in the study are included in the article/**Supplementary Material**. Further inquiries can be directed to the corresponding authors.

## Author contributions

LJZ and XLC designed experiments. XJW, LYZ, SSY, YL, YYR and ZJZ performed experiments. XJW, LJZ, SSY and ZRH analyzed data. XLC, LYZ, and LJZ wrote the manuscript. All authors contributed to the article and approved the submitted version.

## Funding

This work was funded by the Natural Science Foundation of China (31260283 and 31560078) and the Natural Science Foundation of Yunnan Province (NO.202001BB050011) to XLC.

## Acknowledgments

We appreciate Fred Sack (University of British Columbia) for sharing *tmm-1*, *spch-1*, *epf*2, *yda-2, pTMM:TMM-GFP*, *pSPCH:SPCH-GF*P, *pFAMA:FAMA-GFP, pEPF1:GFP*, and *pEPF2:GFP*, the Nottingham Arabidopsis Stock Centre (NASC) for providing *hy1-100* (CS236), *er-105* (SALK_061943), *erl1* (SALK_081669), and *erl2* (SALK_007643), and Gang Liang (Xishuangbanna Tropical Botanical Garden, Chinese Academy of Sciences) for *35S:HY1*.

## Conflict of interest

The authors declare that the research was conducted in the absence of any commercial or financial relationships that could be construed as a potential conflict of interest.

## Publisher’s note

All claims expressed in this article are solely those of the authors and do not necessarily represent those of their affiliated organizations, or those of the publisher, the editors and the reviewers. Any product that may be evaluated in this article, or claim that may be made by its manufacturer, is not guaranteed or endorsed by the publisher.

## Supplementary material

Supplementary Figure 1 Fe-EDTA and BV have no effect on the stomatal development of wild type. **(A)** DIC images of the abaxial cotyledon epidermis of 14d old Wild–type (Col-0) plants with or without Fe-EDTA (40 μM), BV (20 μM) and CORM-2 (40 μM). Bars = 50 μm. **(B)** SIs of Wild–type (Col-0) plants with or without Fe-EDTA (40 μM), BV (20 μM) and CORM-2 (40 μM) treatments. Error bars show standard deviation (SD) (n = 8). Statistical significance of different results was analyzed using Student’s *t*-test: ns Difference is not significant (*p*-value > 0.05). **** Difference is highly significant (*p*-value < 0.0001).

Supplementary Figure 2 Expression of *EPF1* is not affected by CO. **(A)** Confocal images of the abaxial cotyledon epidermis of 4 d old Wild–type (Col-0) harboring *pEPF1:GFP* with or without CORM-2 (20 μM) and ZnPPIX (10 μM) treatment. Bars = 50 μm. **(B)** Number of GFP-positive cells on the cotyledon epidermis of 4d old Wild–type (Col-0) plants expressing *pEPF1:GFP* with or without CORM-2 (20 μM) and ZnPPIX (10 μM) treatment. Error bars show standard deviation (SD) (n = 5). **(C)** Expression of *EPF1* in Wild–type (Col-0) and *hy1-100* with or without CORM-2 (20 μM) and ZnPPIX (10 μM) treatment. Error bars show standard deviation (SD) (n = 3). Statistical significance of different results was analyzed using Student’s *t*-test: ns Difference is not significant (*p*-value > 0.05).

Supplementary Table 1 Primers used in this study.

## Abbreviations

bHLH, basic Helix-Loop-Helix; BV, Biliverdin IXa; CO, Carbon monoxide; CORM-2, Tricarbonyldichlororuthenium (II) dimer; EPFs, Epidermal Patterning Factors; ER, ERECTA; GC, Guard Cell; GMC, Guard Mother Cell; HO, Heme Oxygenase; MAPK, Mitogen-Activated Protein Kinase; MMC, Meristemoid Mother Cell; SI, Stomatal Index; SLGC, Stomatal Lineage Ground Cell; SPCH, SPEECHLESS; TMM, TOO MANY MOUTHS; YDA, YODA; ZnPPIX, Protoporphyrin IX zinc (II).
