# Supplementary figures and images for: Carbon monoxide promotes stomatal initiation by regulating the expression of two EPF genes in Arabidopsis cotyledons
Source: Front Plant Sci. 2022 Nov 11;13:1029703. doi: 10.3389/fpls.2022.1029703 (PMC9691970; doi:10.3389/fpls.2022.1029703)

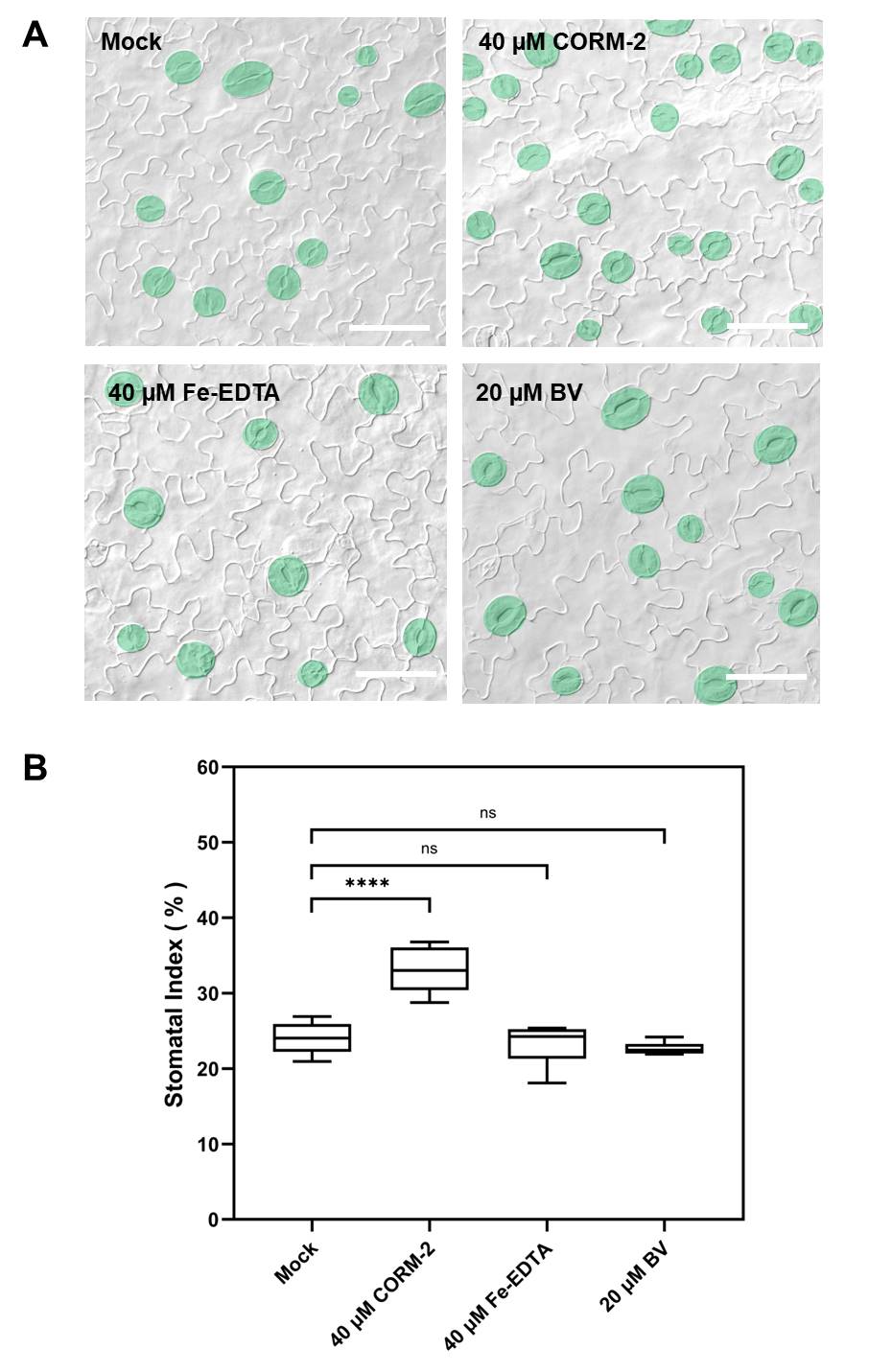

Supplement: Supplementary Figure 1 — Fe-EDTA and BV have no effect on the stomatal development of wild type. (A) DIC images of the abaxial cotyledon epidermis of 14d old Wild–type (Col-0) plants with or without Fe-EDTA (40 μM), BV (20 μM) and CORM-2 (40 μM). Bars = 50 μm. (B) SIs of Wild–type (Col-0) plants with or without Fe-EDTA (40 μM), BV (20 μM) and CORM-2 (40 μM) treatments. Error bars show standard deviation (SD) (n = 8). Statistical significance of different results was analyzed using Student’s t-test: ns Difference is not significant (p-value > 0.05). **** Difference is highly significant (p-value < 0.0001). [file Image_1.jpeg]

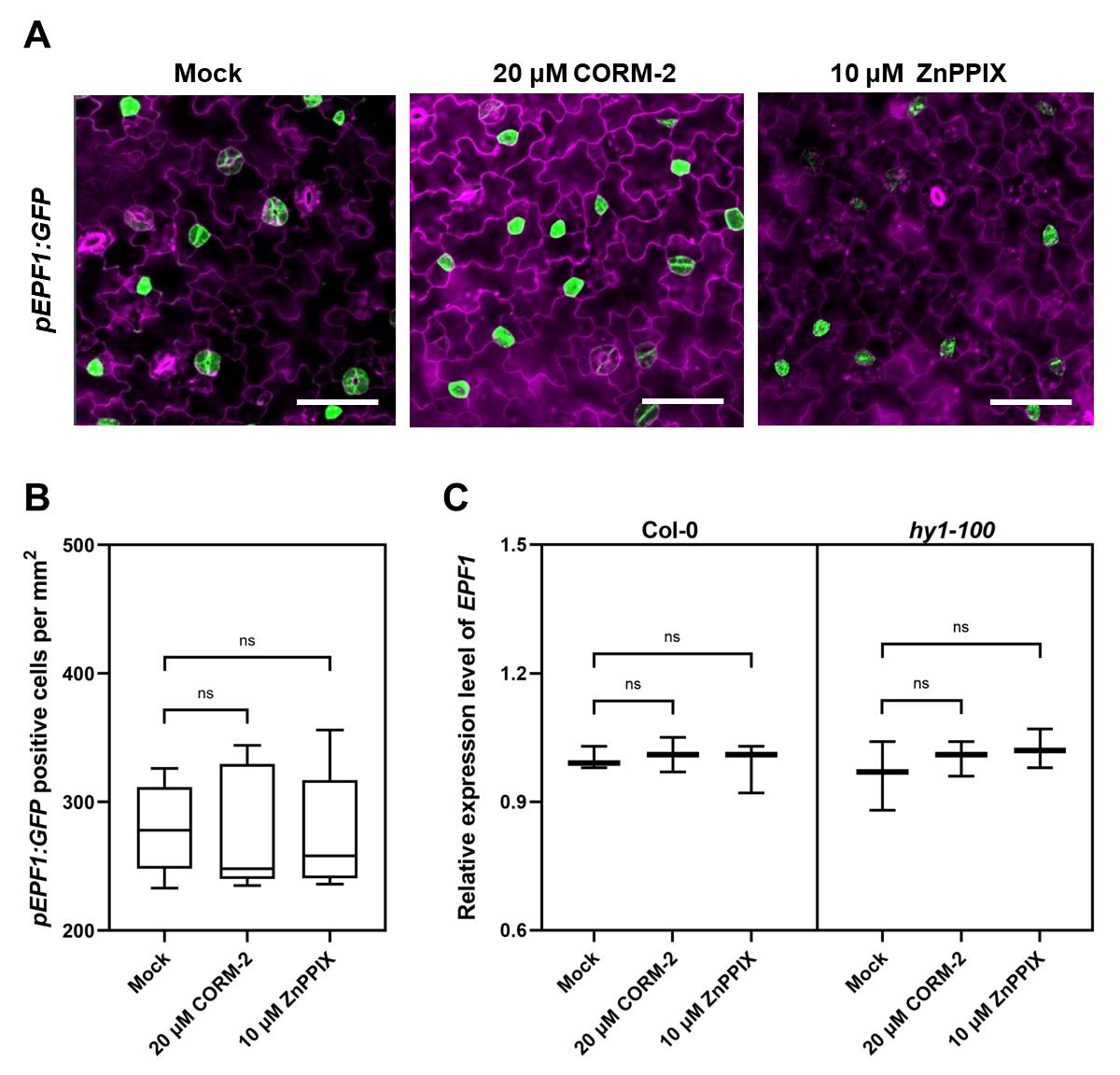

Supplement: Supplementary Figure 2 — Expression of EPF1 is not affected by CO. (A) Confocal images of the abaxial cotyledon epidermis of 4 d old Wild–type (Col-0) harboring pEPF1:GFP with or without CORM-2 (20 μM) and ZnPPIX (10 μM) treatment. Bars = 50 μm. (B) Number of GFP-positive cells on the cotyledon epidermis of 4d old Wild–type (Col-0) plants expressing pEPF1:GFP with or without CORM-2 (20 μM) and ZnPPIX (10 μM) treatment. Error bars show standard deviation (SD) (n = 5). (C) Expression of EPF1 in Wild–type (Col-0) and hy1-100 with or without CORM-2 (20 μM) and ZnPPIX (10 μM) treatment. Error bars show standard deviation (SD) (n = 3). Statistical significance of different results was analyzed using Student’s t-test: ns Difference is not significant (p-value > 0.05). [file Image_2.jpeg]
