# Supplementary material for: Carbon monoxide promotes stomatal initiation by regulating the expression of two EPF genes in Arabidopsis cotyledons
Source: Front Plant Sci. 2022 Nov 11;13:1029703. doi: 10.3389/fpls.2022.1029703 (PMC9691970; doi:10.3389/fpls.2022.1029703)
Supplement: Supplementary Table 1 — Primers used in this study. [file Table_1.docx]

**Supplimentary Table 1. Primers used in this study.**

**Primes used in vector construction:**

*pSTOMAGEN:nucGFP：*

F: gaaagaattcgagctcgcccgggATGAAGCATGAAATGATGAAC

R:ccttgctcaccatggttctagagTTATCTATGACAAACACATCTATA

**Primer used in CRISPR-Cas9 of *STOMAGEN*:**

AtU3b-up-R: TCGACTGGAACTTGCTCTGCTGACCAATGTTGCTCCCTC

AtU3b-down-F: GCAGAGCAAGTTCCAGTCGAGTTTTAGAGCTAGAAATAGCAA

**Primes used in Q RT-PCR**

ACTIN2 ：

F：GATGAGGCAGGTCCAGGAATC

R: AACCCCAGCTTTTTAAGCCTTT

qSTOMAGEN：

F: AGTTCAAGCCTCAAGGACCTCG

R: AACTTGCTCTGCTGCTCTGCACT

qEPF1 ：

F: CATCCTCCCATCCAAGTCATC

R: AGCAATCTGGCAACCTAGAC

qEPF2 ：

F: CGCACCACAAGAAGGAAATAA

R: CCACACGCGTATGAACAATC
